# Differential neural coordination of bilateral hand and finger movements

**DOI:** 10.14814/phy2.14393

**Published:** 2020-03-20

**Authors:** Paolo Caldelari, Roger Lemon, Volker Dietz

**Affiliations:** ^1^ Spinal Cord Injury Center University Hospital Balgrist Zürich Switzerland; ^2^ Queen Square Institute of Neurology University College London London UK

**Keywords:** bilateral hand/finger movements, contralateral reflex activity, forearm muscle activity, neural coupling

## Abstract

Cooperative hand movements (e.g., opening a bottle) require a close coordination of the hands. This is reflected in a neural coupling between the two sides. The aim of this study was to investigate in how far neural coupling is present not only during bilateral hand but also during bilateral finger movements. For this purpose unilateral mechanical and electrical nerve stimuli were delivered during bilateral sequentially and synchronously performed finger movements on a keyboard and, for comparison, during bilateral hand flexion movements. Electromyographic (EMG) activity and reflex responses in forearm flexor and extensor muscles of both sides were recorded and analyzed. Confounding EMG activity related to hand movements during the finger task was limited by wrist fixating braces. During the hand flexion task, complex reflex responses appeared in the forearm muscles of both sides to unilateral stimulation of the ulnar nerve (mean latency 57 ms), reflecting neural coupling between the two hands. In contrast, during the bilateral finger movement task, unilateral electrical nerve or mechanical stimulation of the right index finger was followed by dominant ipsilateral reflex responses (latency 45 and 58 ms, respectively). The results indicate that in contrast to the coupled hand movements, finger movements may not be coupled but can move independently on each side. Functionally this makes sense because during most activities of daily living, a close cooperation of the hands but not of individual fingers is needed. This independence of individual finger movements may rely on strong, specific, contralateral cortico‐motoneuronal control.

## Graphical abstract

The coordination of bilateral hand movements is reflected by a neural coupling. In contrast bilateral finger movements can move independently on each side. This independence of finger movements may rely on strong, specific, contralateral cortico‐motoneuronal control.

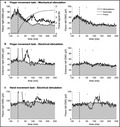

## 1 INTRODUCTION

Research on neural hand motor control is focused mainly on unilateral reach and grasp movements. However, a broad spectrum of bimanual movements is also needed for activities of daily living (ADL). For example, cooperative hand movements are required when opening a bottle, that is, the action of the two hands is different, but they support each other. Previous research on cooperative hand movements indicated that neural coupling between the two hands provides a fast and accurate automatic coordination of bilateral hand movements in ADL (Dietz et al., 2015). This neural coupling is reflected in the appearance of bilateral reflex EMG responses to unilateral arm nerve stimulation. The size of reflex responses was shown to depend on the level of forearm muscle activity, that is, an increase in movement velocity or resistance results in greater background muscle activation and, consequently, in greater reflex amplitudes not only ipsi‐ but also contralateral to the stimulation site (Thomas, Dietz, Scharfenberger, & Schrafl‐Altermatt, 2018).

This reflex behavior corresponds to that described earlier showing that the size of ipsilateral responses to a perturbation is related to the level of background EMG activity (Matthews, 1986). The fact that this behavior also applies to the contralateral reflex response during bilateral hand movements suggests an automatic gain control of neural coupling, allowing a rapid adaptation of the forces exerted by the hands on an object.

The aim of this study was to compare the control of hand and finger movements. Little is known about the neural coordination of bilateral finger movements. Unilateral hand and finger movements are important for reaching, grasping, and object manipulation. The safe performance of such unilateral movements is based on a fast compensation of unexpected finger/hand perturbations by the activity of polysynaptic, long‐latency reflexes (Cole, Gracco, & Abbs, 1984; Marsden, Merton, & Morton, 1976; Matthews, 1986) in combination with a close interaction between hand and finger muscles (Lemon, Johansson, & Westling, 1995). In this context it could be hypothesized that the neural coupling mechanism coordinates not only bilateral hand but also bilateral finger movements. Such a coupling could be expected during bilateral finger movements on the basis of observations made in young children (Mayston, Harrison, & Stephens, 1999) and some movement pathologies (e.g., Kallmann's syndrome, cf. [Mayston et al., 1997]). Thus, the fact that the basic circuitry for a neural coupling of bilateral finger movements seems to be available it makes sense to explore the presence of bilateral reflexes to unilateral perturbations. However, the results presented here suggest that during finger movements, the hands and fingers are differentially controlled, and bilateral reflex activity is absent in finger muscles.

## 2 METHODS

Seventeen participants with a mean age of 27.1 ± 3.7 years (9 male, 8 females; 15 right‐ and 2 left‐handed: self‐reported) were recruited for the study. Written consent of the participants was obtained before starting the experiment. The study was approved by the Ethics Committee of the Canton Zürich.

### 2.1 Experimental design

**FIGURE 1:**
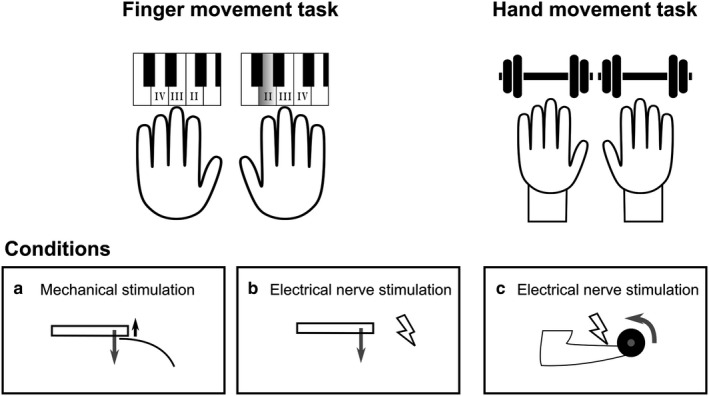
Illustration of the experimental tasks and conditions. The experiment consisted of two tasks. One task, consisted of bilateral synchronously performed sequential finger tapping movements on a keyboard, included two conditions. These conditions differed in the type of randomly applied dummy or experimental stimuli which were released when the prepared key was touched by the right index finger. In condition a. ‘mechanical’: stimulation an increased resistive force of the key occurred (upward arrow in bottom left inset) against the downward movement of the key by the index finger. During the second condition b. ‘electrical’: the initiation of key press by the right index finger randomly triggered the delivery of an electrical stimulus to the right ulnar nerve. The second task c. consisted of bilateral hand flexion movements while holding dumbbells (load: 0.5 kg). Electrical stimuli to the ulnar nerve were randomly applied during the rising phase of the movements

A specific, custom built, experimental approach was developed in order to assess the coordination of bilateral finger movements and to compare it with that of bilateral hand movements, that is, whether reflex responses suggest that neural coupling exists not only between the hands but also between the fingers. The experiment comprised two tasks: a "finger movement task" that included two conditions; and a "hand movement task" with one condition. An overview of the tasks and conditions is shown in Figure 1. Following instruction of the tasks, the participants were allowed two minutes of practice for each task and were randomly allocated to start with one of the two tasks. The two conditions of the finger task were performed in a random order to minimize habituation to the mechanical and/or electrical nerve stimuli which were randomly delivered during the different movement conditions. Breaks of five minutes were introduced between the conditions to prevent fatigue.

### 2.2 Finger movement task

The “finger movement task” (Figure 1) consisted of a defined finger tapping sequence performed on a keyboard (Technics, SX KN901, Japan). The key tapping of the fingers II‐IV was performed sequentially and bilaterally in synchrony. The key strike frequency of 1.33 Hz (80 beats per minute), was provided by the acoustic signal of a metronome. Participants were instructed to keep their wrists in a neutral position (in line with the forearms) and their fingers extended. In addition braces were applied to the forearms and hands to minimize wrist movements during the finger task. The instruction was to press the keys dynamically by flexing the fingers II to IV sequentially at the metacarpo‐phalangeal joints. Mechanical and electrical stimuli were randomly delivered at the onset of key press by the right index finger.

The two stimulation conditions in the finger task consisted of (Figure 1a) an increase in key resistance when the key was pressed by the right index finger (mechanical) and, (Figure 1b) non‐noxious electrical stimulation of the right ulnar nerve triggered by the contact of the key with the finger (electrical).

### 2.3 Preparation of a key for mechanical single finger stimulation

**FIGURE 2:**
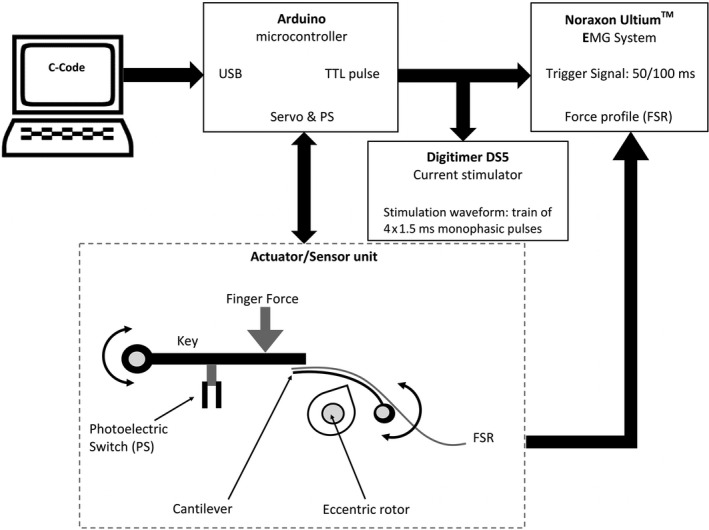
Block diagram illustrating the technical design of a single keyboard key which was prepared for mechanical finger stimuli and for triggering mechanical and electrical stimuli. The custom‐built setup was placed under the key which was to be pressed by the right index finger. The design enabled the random delivery of (a): mechanical stimuli, (b): stimuli for triggering the EMG responses to mechanical/electrical stimuli. To implement these functions an Arduino microcontroller was used. The C‐code used to program the Arduino microcontroller was compiled with the Arduino IDE Software. The onset of the downward movement of the key activated the photoelectric switch which then started the random generator controlled by the Arduino. The random generator released mechanical/ electrical stimuli or no stimuli depending on the experimental condition. For the mechanical perturbation, the servo pressed the cantilever against the bottom of the key to return the key after the initial downward movement of the key by the subject's right index finger. For electrical stimuli, the TTL‐pulse was used to trigger electrical stimuli delivered by a Digitimer DS5 and triggering of the EMG responses for electrical or dummy stimuli. C‐code: C programming language used by Arduino Uno microcontroller; USB: Universal Serial Bus; interface between laptop and Arduino; TTL pulse: Transistor Transistor Logic pulse (5 V) generated by Arduino; FSR: Force Sensing Resistor; sensor for the force measurement between cantilever and the key; Servo & PS: Interface between Arduino and the Actuator/Sensor unit. This interface had two functions: Steering the servo motor and reading of the PS (Photoelectric Switch) sensors

For the application of unilateral mechanical and electrical stimuli and for triggering reflex EMG responses in the forearm muscles in the finger movement task, one key of the keyboard was adapted (for technical details see legend to Figure 2). In order to increase the natural key resistance force from 1 N to about 3 N, a servo motor actuator with an integrated gear was employed. A microcontroller board was used for precision control of the servo motor. The maximum elastic resisting force *F* = (0.16 N/mm × 12 mm) + 1 *N* = 2.9 N of the key was achieved against a 10 mm downward finger movement, whereas the natural key resistance amounted to about 1 N.

The prepared key was equipped with a tactile sensor consisting of a force sensing resistor (FSR) (Model 402, 0.5, “INTERLINK Electronics”). The FSR was directly fixed to the cantilever. The force signal recorded by the FSR was transferred in real time using the EMG device from Noraxon. A photoelectric switch (Figure 2) served as a sensor, to generate different TTL‐triggered pulses (TTL = Transistor Transistor Logic), in order to randomly produce the appropriate trigger signal for recording the forearm reflex EMG activity during the different experimental conditions. A 50 ms pulse indicated a key movement without stimulation, whereas a 100 ms pulse indicated the delivery of a stimulus. For electrical stimuli, the TTL‐pulse was used to define stimulus delivery (see below “electrical ulnar nerve stimulation”).

### 2.4 Hand movement task

The reflex behavior during bilateral finger movements was compared with that previously described for bilateral hand movements (Thomas, Dietz, Scharfenberger, et al., 2018). This task consisted of bilateral synchronous palmar hand flexion movements at a frequency of 1.33 Hz (acoustic signal of the metronome) while holding 0.5 kg dumbbells. Palmar hand flexion movements were chosen to be comparable to the flexion movement of the fingers during the finger task. Participants were instructed to place the supinated forearms with the wrists extended over the table top. The ulnar nerve was randomly stimulated during the rising phase of the hand movements (Figure 1c).

### 2.5 Electromyographic recordings

EMG activity and reflex responses were recorded for both finger and hand movement tasks over the forearm extensor and flexor muscles of both arms using pairs of hydrogel knob surface electrodes (KendallTM H124SG = 2,4 cm diameter). Signals were sampled at a rate of 2,000 Hz and recorded using a wireless system (Noraxon). Postprocessing with Matlab R2017b included offset correction, bandstop filtering between 45 and 55 Hz and rectification of the signal (for further details see Dietz et al. (2015); Thomas, Dietz, and Schrafl‐Altermatt (2018).

### 2.6 Electrical ulnar nerve stimulation

For the conditions using electrical nerve stimulation, non‐noxious stimuli were transcutaneously applied to the ulnar nerve through the overlying skin (using a DS5 isolated bipolar constant current stimulator (Digitimer) through self‐adhesive surface electrodes (Ambu® A/S Neuroline 700). Stimulation electrodes (inter‐electrode distance 2 cm, cathode proximal) were placed just proximal to the wrist crease over the distal ulnar nerve. During the finger task, a total of 15 trains of stimuli were randomly delivered at the start of the downward movement of the key by the right index finger. For the hand movement task, the 15 stimuli were randomly delivered during the rising phase of the hand flexion movements. The interval between consecutive stimuli was set to at least 3 s for both the finger and the hand movement tasks. The trigger was generated by the Arduino microcontroller and consisted of a TTL pulse width of 12 ms.

The DS5 current stimulator delivered a stimulus proportional to the input analogue voltage generated by the Arduino. Since the Arduino Uno output was only able to deliver a voltage of 5 V, a variable attenuator had to be interposed in order to deliver the stimulation current that was needed to evoke the defined stimulation intensity. Furthermore, the Arduino Uno allowed only to generate monophasic pulses. This differs from a previous study that used a train of four biphasic pulses (Thomas, Dietz, & Schrafl‐Altermatt, 2018). Therefore, the generation of electrical stimuli was adapted in a way which allowed to apply similar stimulation intensities. Each stimulus consisted of a train of four monophasic pulses (duration 1.5 ms), separated by 2 ms, with a total stimulus duration of 12 ms. The stimulation intensity was set at 150% of motor threshold. Motor threshold was defined as the first visible twitch of the M. abductor digiti minimi. “Dummy” trials were randomly interleaved with trials in which stimuli were delivered; around 75% of trials were dummy trials.

### 2.7 Data analysis

The latencies of the reflex responses in both tasks were determined by subtracting the responses from the background EMG. Because the configuration of the reflexes evoked during finger and hand tasks differed in shape and latency, no quantitative comparison between tasks was made.

For the quantitative analysis of the reflex responses to stimulation and the background EMG activity recorded during “dummy” trials, root mean squares (RMS) were calculated. The RMS of the rectified EMG signals was calculated for each task and condition in a defined time window after stimulation onset (see below). Rectification of the EMG signals of background and reflex activity was performed for calculation of RMS values in order to include also negative components of the reflex responses. The mean of the background EMG signal in the defined time window was used as the baseline for signal rectification.

For the analysis of the finger movement task the time window was adapted to include the main reflex component. The mechanical stimuli were followed by reflex responses with longer latencies and durations compared with those to electrical stimuli. Therefore, a time window of 50–135 ms was chosen for the reflex analysis after mechanical stimulation. The reflex response to electrical stimulation appeared earlier and its duration was shorter compared with those to mechanical stimuli. Therefore, a time window between 40 ms and 90 ms was chosen.

For the analysis of the hand movement task, a time window between 50 ms and 135 ms after stimulation onset was chosen according to other studies (Dietz et al., 2015) and (Thomas, Dietz, & Schrafl‐Altermatt, 2018). This time window comprises the negative and positive reflex components.

To quantify the size of the reflex response the mean of the absolute differences between the reflex responses and the dummy trial EMG signal in their respective time window was calculated. This calculation was performed for all three conditions to compare the reflex responses on the ipsilateral versus the contralateral side.

### 2.8 Statistical analysis

Both the RMS values and the means of the absolute reflex responses were not normally distributed according to the Shapiro–Wilk test. Hence a nonparametric test namely the two‐sided Wilcoxon signed rank test was selected to test differences in either variable. First, for each experimental condition, it was tested whether the RMS values of the stimulated trials differed from their respective background EMG during the dummy trials. This was performed in order to confirm the presence of a reflex response. Second, ipsi‐ and contralateral reflex responses were compared in order to assess neural coupling.

The *p* values <.05 were considered significant. RMS values as well as the mean of the absolute reflex response are given as median and interquartile range (IQR, 25th–75th percentile).

## 3 RESULTS

Seventeen volunteers participated in the study and were involved in all experimental conditions. Nevertheless, several recordings had to be excluded due to technical problems. Overall in the finger movement task 15 recordings with mechanical stimuli and 12 recordings with electrical stimuli were further analyzed. In the hand movement task 13 recordings were further analyzed.

**FIGURE 3:**
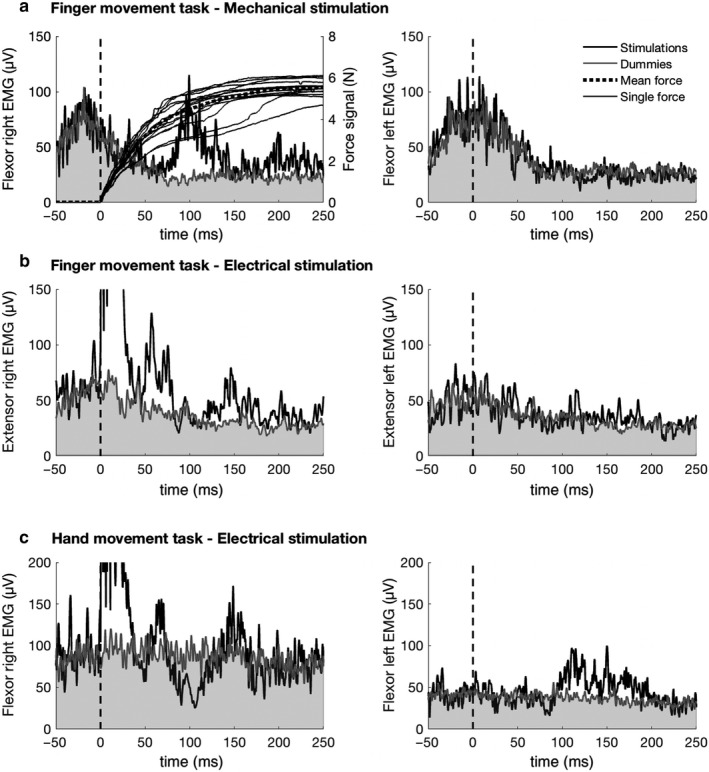
Bilateral forearm muscle EMG activity and reflex recordings of the two experimental conditions in the finger task (a: mechanical, b: electrical stimuli) and of the hand task (c) from one representative subject. Fifteen stimuli were applied in every condition. In (a) the averaged, rectified EMG recordings of the forearm flexors is shown together with the single trial and average (thick line) force signals after mechanical stimuli. In (b), responses of the extensors of both sides following electrical stimuli are illustrated. During the hand task (c) the flexor muscles were activated. Vertical dotted line indicates onset of stimulation; the gray areas reflect the EMG activity following dummy trials without stimuli

In Figure 3 typical averaged, rectified EMG activity of forearm muscles of both sides from one subject is shown. The reflex responses to unilateral right‐sided mechanical (Figure 3a) and electrical (Figure 3b) stimuli during the finger task are displayed. For comparison, responses to right‐sided electrical stimuli during the bilateral hand task (Figure 3c) are also shown. For each condition an average of the EMG responses to 15 trains of stimuli was calculated. The vertical dashed line indicates the onset of stimulation. Stimulation artifacts associated with ulnar nerve stimuli lasted over 17ms. The gray area represents nonstimulated (dummy) trials. Figure 3a also shows the force traces from single trials recorded during perturbation of the downward movement of the right index finger. Both mechanical and electrical stimuli were followed by reflex responses which were restricted to the ipsilateral side, and which appeared in the forearm flexor muscles with a latency of 57 ms (Figure 3a, mechanical) and in forearm extensor muscles with a latency of 45 ms (Figure 3b, electrical). For comparison, electrical stimuli delivered during the hand task were followed by complex reflex responses which appeared in the activated forearm flexor muscles of both sides with a latency of approximately 65 ms (right side 62 ms, left side 64 ms; Figure 3c). The smaller amplitude of the contralateral reflex response can be attributed to the smaller background EMG activity (Thomas, Dietz, & Schrafl‐Altermatt, 2018).

**FIGURE 4:**
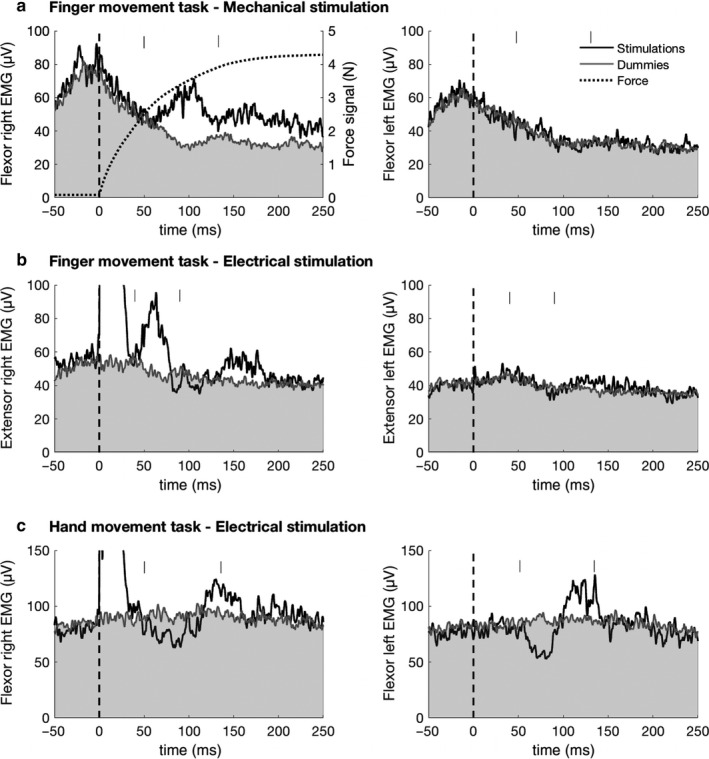
Grand averages of bilateral forearm muscle EMG activity with reflex responses of the two experimental conditions in the finger task (a, mechanical (*n* = 15 subjects) and b, electrical stimuli (*n* = 12 subjects)) and for comparison of the hand task (c, *n* = 13 subjects). Gray areas reflect the EMG activity in dummy trials without stimuli. The vertical lines indicate the windows used for the quantification of the reflex responses on both sides. In (a), the dotted line represents the averaged force signals after mechanical stimuli

Figure 4 shows the grand averages of the recordings with the appearance of unilateral and bilateral reflex responses in different forearm muscles in the three experimental conditions (Figure 4, a (*n* = 15), b (*n* = 12), c (*n* = 13)). During bilateral finger movements, mechanical stimuli to the right index finger (Figure 4a) were followed by reflex responses which appeared in the ipsilateral forearm flexor muscles (latency approximately 58 ms, duration approximately 80 ms). The dotted line indicates the average force produced by the key against the downward movement of the right index finger. Right ulnar nerve stimuli (Figure 4b) were followed by distinct ipsilateral responses in the right extensor muscles (latency measured from the first stimulus approximately 45 ms, duration approximately 50 ms). In both conditions of the finger task, reflex responses on the contralateral side were either small (Figure 4b) or absent. Right ulnar nerve stimuli during bilateral hand flexion movements while holding dumbbells of 0.5 kg (Figure 4c) were followed by complex reflex responses which appeared in the forearm flexor muscles on both sides (latency approximately 57 ms; duration approximately 130 ms). In the other forearm muscles, not shown in the figure (a extensors, b flexors, c extensors), no reflex responses appeared and they were therefore not included in the following quantitative analysis.

**FIGURE 5:**
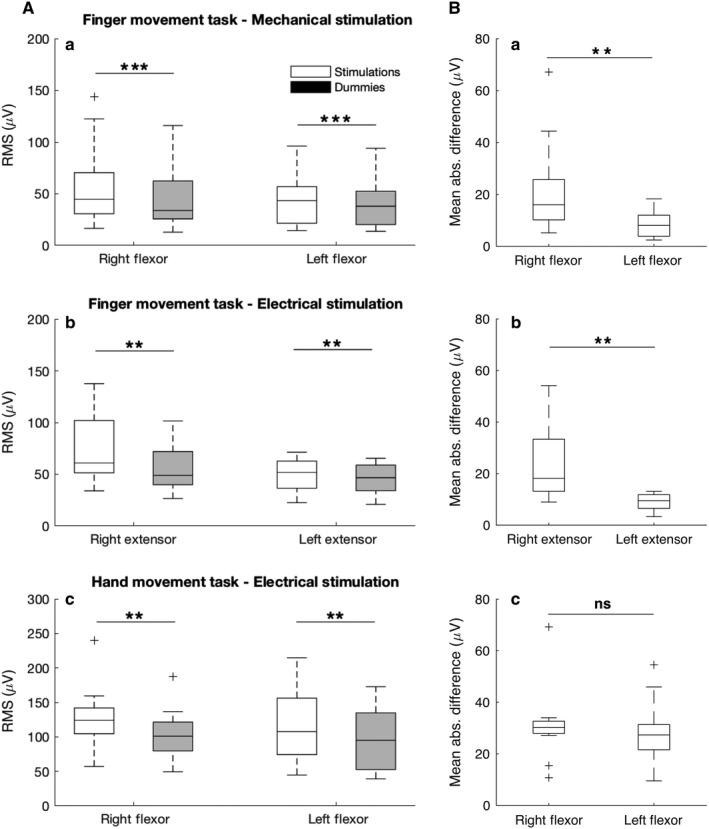
(A) Quantified RMS values of reflex responses and background EMG (dummy trials) calculated for the time windows indicated in Figure 4. Dummy trials are represented by gray bars and experimental conditions by white bars. There was a significant difference between RMS values of both ipsilateral and contralateral reflex responses to mechanical (Aa; *n* = 15 subjects) and electrical (Ab; *n* = 12 subjects) stimuli for the finger task as well as for the hand task (Ac; *n* = 13 subjects) compared with the RMS of background EMG activity following dummy stimuli. (B) Absolute reflex responses for the ipsilateral and contralateral side: Mean absolute difference values were calculated as the absolute difference between reflex responses to mechanical (Aa) and electrical (Ab) stimuli for the finger task as well as for the hand task (Ac) and the background EMG activity in dummy trials. Ipsilateral reflex responses where larger than contralateral responses to unilateral (right side) mechanical (Ba) and electrical (Bb) stimuli in the finger movement task but not for the hand task (Bc). Ba; *n* = 15 subjects, Bb; *n* = 12 subjects, Bc; *n* = 13 subjects). Significant differences in (A) and (B) are indicated by one asterisk (*p* < .05), two asterisks (*p* < .01), or three asterisks (*p* < .001)

Figure 5A,B summarizes the quantitative data for the three experimental conditions (a (*n* = 15), b (*n* = 12), c (*n* = 13). In Figure 5A EMG background activity (dummy trials) was compared with EMG responses in the two conditions of the finger task with mechanical (Figure 5Aa) and electrical (Figure 5Ab) stimuli. Reflex responses during the hand task are shown in Figure 5Ac. For all experimental conditions (5Aa‐c) the reflex RMS responses after stimulation were larger compared with the background RMS obtained in the dummy trials. This was the case for muscles of both ipsilateral and contralateral to the stimulation side.

Following mechanical stimuli (Figure 5Aa), the ipsilateral (right flexor) reflex response RMS values (median 44.67, IQR 30.55–73.14 µV) were significantly larger compared with background EMG values (33.93, 25.05–65.70 µV) (T = 0, *p* = .0007, *r* = .62, *n* = 15). A similar difference was also present for the contralateral (left flexor) reflex response where RMS values (43.32, 21.41–57.57 µV) were significantly larger compared with the background EMG (37.96, 20.19–52.81 µV) (T = 0, *p* = .0007, *r* = .62, *n* = 15).

Following electrical stimuli (Figure 5Ab) RMS values were significantly larger for responses in the ipsilateral extensor muscles (60.90, 50.25–112.80 µV) compared with the background RMS values (48.97, 39.68–73.09 µV) (T = 0, *p* = .0022, *r* = .62, *n* = 12). Once again, this was also true for the contralateral extensor RMS value (51.79, 35.81– 62.84 µV) compared with the background RMS (46.71, 33.9–59.57 µV) (T = 0, *p* = .0022, *r* = .62, *n* = 12).

For comparison, during the hand task (Figure 5ac), reflex response RMS values on the ipsilateral side (right flexor), (123.93, 103.60–143.76 µV) were greater than dummy trial RMS values (101.01, 77,63–122.07 µV) (T = 0, *p* = .0015, *r* = .62 *n* = 13). The same was true for the contralateral side, with RMS values (107.78, 72.12–157.32 µV) larger than background RMS values (94.96, 51.62–137.10 µV) (T = 0, *p* = .0015, *r* = .62 *n* = 13).

In Figure 5B, the mean absolute reflex responses were compared between the ipsilateral and contralateral sides. For the finger task, a two‐sided Wilcoxon signed rank test revealed that following mechanical stimuli (Figure 5Ba) absolute reflex values were significantly greater on the ipsilateral (stimulated) side (16.00, 9.96–26.43 µV) compared with the contralateral (nonstimulated) side (8.04, 3.75–12.06 µV) (T = 9.00, *p* = .0038, *r* = .53, *n* = 15). Correspondingly, following electrical stimuli (Figure 5bb) reflex responses on the ipsilateral side were greater (18.18, 12.29–35.03 µV) compared with the contralateral side (9.45, 6.18–11.96 µV) (T = 0.00, *p* = .0022, *r* = .62, *n* = 13). In contrast, during the hand movement task, (Figure 5Bc) reflex responses did not differ significantly between the ipsilateral (30.26, 27.67–32.74 µV) and contralateral side (27.31, 20.41–31.47 µV) (T = 39, *p* = .6496, *r* = .09, *n* = 13).

## 4 DISCUSSION

This study deals with the question of whether the neural coupling associated with bilateral hand movements is also present during bilateral finger movements. This neural coupling is reflected in the appearance of reflex responses in the forearm muscles of both sides to unilateral ulnar nerve stimulation (Dietz et al., 2015). On the basis of the close interaction of hand and fingers during reach and grasp movements (Lemon et al., 1995) and the observations made in young children (Mayston et al., 1999) and some movement pathologies (Mayston et al., 1997) one could hypothesize that bilateral neural coupling of the fingers might also be present.

The appearance of the reflex responses to mechanical and electrical stimuli in the forearm flexors and extensors, respectively, is assumed to be due to the different modes of stimulation. That is, stretch of the finger flexor muscles and activation of skin mechanoreceptors following the mechanical stimulus, and activation of cutaneous and proprioceptive afferents by electrical stimulation of the ulnar nerve, respectively. We took care to separate wrist from finger movements by the application of wrist fixating braces. Thus, we suggest that under this condition EMG activity was recorded from long finger flexors (or extensors) in the finger task but from the wrist flexors (or extensors) in the hand task. Nevertheless, independently of the muscles activated during the two tasks, the important observation concerns the fact that they were differentially controlled.

The main results obtained were: (a) In contrast to the presence of neural coupling during bilateral hand movements, contralateral reflex responses were small or absent during bilateral finger movements, and this was true for both unilateral mechanical and electrical stimuli. This suggests that neural coupling of the two sides is virtually absent during bilateral finger movements. (b) The ipsilateral reflex responses to mechanical/electrical stimuli differed in latency and shape from the bilateral reflex responses evoked during bilateral hand movements. These results will be discussed with regard to functional and neuro‐anatomical implications.

### 4.1 Task‐dependency of neural coupling

Our findings are in line with previous findings showing a neural coupling during cooperative and noncooperative bilateral hand movements (Dietz et al., 2015; Thomas, Dietz, & Schrafl‐Altermatt, 2018). In contrast, from the finger movement task studied here it appears that there is no reflex coupling between the fingers of both sides. Therefore, we suggest that the control of skilled finger movements, as here in keyboard playing, is carried out separately for the two sides, possibly reflecting the highly crossed nature of cortico‐motoneuronal connections to digit muscles (Lemon, 2008; Morecraft et al., 2013). The capacity to perform skilled hand/finger movements has been suggested to rely on the evolutionary formation of these direct cortico‐motoneuronal projections to the alpha motoneurons of hand and finger muscles (Kuypers, 1981; Lemon, 2008). These projections originate in the “new M1” region of the primary motor cortex and are restricted to humans and dexterous primates (Rathelot & Strick, 2009). These projections are almost exclusively contralateral (Morecraft et al., 2013).

The differential neural control of bilateral hand and finger movements may be due to their different function in activities of daily life (ADL). For most of these activities the neural coupling of bilateral hand movements is functionally meaningful and in these conditions fingers are used as part of the hand. Correspondingly, during object manipulation involving the hands and fingers of both sides, grip force responses to perturbations were shown to be linked (Bracewell, Wing, Soper, & Clark, 2003; White, Dowling, Bracewell, & Diedrichsen, 2008).

In contrast, when bilateral single finger movements are performed in isolation, they may move independently on the two sides, in a noncoupled manner. The basic circuitry for a neural coupling of the fingers might be available in principle cf. (Mayston et al., 1997) but is possibly suppressed in healthy adults. Although long‐latency reflexes still operate ipsilaterally (Evans, Harrison, & Stephens, 1989), coupling of bilateral finger movements through these pathways may not be needed. Temporal coordination of the finger movements on the two sides, as in piano playing, for example, could arise through other feed‐forward pathways linking the cortico‐motoneuronal outputs of the two hemispheres.

### 4.2 Differential reflex behavior

The different reflex behavior characterizing bilateral hand and finger movements concerned not only the bilateral and unilateral appearance of the reflex responses, respectively, but also the fact that both latency and configuration of the reflex responses differed between hand and finger movements. The reflex response during finger movements had a shorter latency and consisted essentially of one component. The somewhat longer latency to mechanical (about 55 ms) compared with electrical (about 45 ms) is assumed to be due to the fact that afferent inputs are generated earlier following electrical stimulation. The ipsilateral reflex response to electrical nerve stimulation in the finger task corresponds to the compensatory reflex responses to unilateral finger stimulation (e.g., Cole et al., 1984; Marsden et al., 1976; Matthews, 1984; Mayston et al., 1999). These responses were suggested to be mediated via a transcortical reflex pathway. Thus there was no difference in reflex behavior between uni‐ and bilateral finger movements, that is, the reflex responses appeared only on the side of stimulation.

In contrast, during the hand movement task, the bilateral reflex response to unilateral electrical stimulation had a complex configuration and appeared with longer latency (about 65 ms). This differential behavior is assumed to reflect the more complex generation of these reflexes for the neural coupling of the hands. The suggested pathways involved include the secondary somatosensory cortices (S2) where shared cutaneous input from both hands converges (Dietz et al., 2015; Goble et al., 2010; Grefkes, Eickhoff, Nowak, Dafotakis, & Fink, 2008). This results in the generation of compensatory responses on both sides to unilateral perturbations with the consequence that bilateral hand movements are mirrored. This coupling may assist the fine coordination of hand movements in daily life activities, such as opening a bottle with the hands while one hand becomes perturbed. Nevertheless, an alternative subcortical origin of mirrored movements caused by the upregulation of bilaterally organized subcortical system (Ejaz et al., 2018) cannot be excluded.

### 4.3 Functional considerations

The observations made suggest a differential neural control of bilateral finger compared with hand movements. This observation makes functional sense in so far that a close cooperation of bilateral hand but not of individual finger movements is needed to tackle many activities of daily living. Independent finger movements in human beings are an essential part of the evolution and represent the basis of human cultural life (Herder, 1785). For example, independent finger movements allow to play piano pieces which require the execution of quavers by the fingers of the right and triplets by those of the left hand (e.g., piano pieces of Debussy). The associated neural coupling of hand movements might be critical for the performance of independent finger movements (e.g., for the preservation of rhythmicity between the two sides during playing piano).

### 4.4 Limitations of the study

First, the EMG recordings from the forearm extensor and flexors muscles through surface electrodes did not allow us to discriminate between finger or hand/wrist muscle activation. Nonetheless, some precautions were applied in order to limit the influence of confounding factors and isolate the result to the finger flexion movement. Namely, the instruction to play with fully extended finger joints and wrists and the application of wrist fixating braces during the finger task. The small contralateral reflex effects seen in some subjects during the finger task, revealed by the statistical analysis (Figures 4b and 5Ba,b), might be due to the fact that associated wrist movements could not be completely prevented by the braces.

Second, for comparison of finger and hand movement task, we studied flexion movements in both cases. This implies that during the finger task the forearms were in a pronated position, but were supinated during the hand task, that is, EMG signals could arise from different muscles due to the change in location of the surface electrodes as the forearm position was changed. However, the same reflex behavior was present in earlier recordings where bilateral hand extensor movements were performed in a pronated position of the forearms (Thomas, Dietz, & Schrafl‐Altermatt, 2018).

Third, the forces and the dynamics of the finger movements exerted by the participants varied in the different conditions. During rhythmic finger tapping, the index finger force depends on handedness with a stronger force exerted by the finger of the dominant hand (Inui & Hatta, 2002). This factor was not controlled in the study.

Fourth, reflex recordings were made over the forearm flexors and extensors at different levels of muscle activation. These factors could affect reflex behavior. In the finger task, due to the different modes of stimulation, reflex responses appeared earlier and in a more synchronized form following electrical stimulation. We suggest this is due to the more synchronized afferent volley excited by electrical stimulation.

Fifth, the slightly larger background activity during the hand compared with the finger task might affect reflex amplitude (Thomas, Dietz, & Schrafl‐Altermatt, 2018). However this difference should not influence the fact that the reflex responses in this task appeared on both sides, while no bilateral reflex responses appeared in the finger tasks.

## CONFLICT OF INTERESTS

The authors declare no competing interests.

## AUTHOR CONTRIBUTIONS

PC performed data acquisition and analysis. He wrote the first draft of the manuscript. RL advised us and revised the manuscript. VD conceived the study, participated in its design, and co‐wrote the manuscript.

## Data Availability

The datasets generated and analyzed during this study are available from the corresponding author on request.
